# Dengue hemorrhagic fever with bleeding and fluid overload in a patient with active lupus nephritis: a case report of diagnostic and therapeutic challenges

**DOI:** 10.1186/s12879-023-08415-5

**Published:** 2023-06-26

**Authors:** Manudi Vidanapathirana, Inoshi Atukorala

**Affiliations:** 1 Professorial Medical Unit, National Hospital of Sri Lanka, Colombo, Sri Lanka; 2 University of Colombo, Colombo, Sri Lanka

**Keywords:** Dengue, Dengue hemorrhagic fever, SLE, Lupus nephritis, Nephrotic syndrome, Acute kidney injury, Fluid management

## Abstract

**Background:**

Diagnosis and management of dengue hemorrhagic fever (DHF) can be challenging in the presence of confounding comorbidities. Important confounders are conditions that alter hematological parameters and intra/extra vascular fluid distribution. We report the case of a patient with active lupus nephritis, who developed DHF with subsequent bleeding and fluid overload. This is the first case report to highlight a unique set of diagnostic and therapeutic challenges in DHF in this context.

**Case presentation:**

A seventeen-year-old girl with lupus nephritis class IV developed a renal flare of lupus and subsequently developed DHF with bleeding per vaginum. Due to acute kidney injury, she was managed with a restrictive fluid approach during the ascending limb with blood transfusion when required, and close monitoring for hemodynamic instability. During the descending limb, hourly input was transiently increased due to a rise in hematocrit. This precipitated nephrogenic pulmonary edema, which was managed with mechanical ventilation and continuous renal replacement therapy.

**Conclusions:**

This patient posed two diagnostic challenges: diagnosis of dengue in a patient with lupus-related bicytopenia, and diagnosis of dengue leakage in a patient with nephrotic syndrome-related ascites. There were three therapeutic difficulties: deciding the fluid quota for DHF in the presence of renal impairment, and balancing the risks and benefits of steroids and anticoagulation in lupus nephritis with dengue. As decisions in such instances are patient-specific, sharing of individual experiences will help guide management decisions.

## Background

Management of dengue fever (DF) and dengue hemorrhagic fever (DHF) has been standardized by increased understanding of its pathophysiology. However, its diagnosis on a background of comorbidities with altered hematologic parameters, and its management in the presence of comorbidities that restrict fluid administration remain a grey area.

We report the case of a patient who posed challenges in both of these contexts. This was a girl with lupus nephritis class IV who initially presented with a renal relapse associated with disease-related bicytopenia. She then developed DHF with bleeding during the ascending limb, and fluid overload during the descending limb.

Systemic lupus erythematosus (SLE) and immunosuppressive medications used in SLE, alter hematological parameters, with potential to confound the diagnosis of DF/ DHF [1]. Further, lupus nephritis(LN) and acute kidney injury (AKI) alter intra/ extravascular fluid balance, making the diagnosis of onset of leakage in DHF and its fluid management challenging.

This case report with explore two diagnostic and two therapeutic challenges posed by this patient under the following themes: First, the diagnosis of dengue in a patient with lupus-related bicytopenia and diagnosis of dengue leakage in a patient with nephrotic syndrome related ascites. Second, deciding on the fluid quota for dengue in the presence of AKI, and balancing the risks and benefits of steroids and anticoagulation in lupus nephritis with dengue. As these decisions are patient-specific, sharing of individual experiences will help further guide management decisions in the future.

## Case presentation

A 17-year-old Sri Lankan girl, recently diagnosed with SLE and class IV lupus nephritis was treated with intravenous (IV) methylprednisolone and cyclophosphamide according to the EuroLupus regime. Four days after the 1st dose of cyclophosphamide, she developed a febrile illness associated with vomiting and worsening of generalized edema.

The patient presented to hospital on the 6th day of fever. Examination at this time revealed gross ascites and bilateral pleural effusions. Her blood pressure was 140/100 mmHg consistent with her baseline blood pressure, and there was no skin rash on presentation. The working diagnosis on initial presentation was a relapse of nephrotic syndrome.

Initial laboratory investigations showed a white blood cell (WBC) count of 9.28 × 10^3^/uL (lymphocyte count-1.92), hemoglobin of 7.7 g/dL and a platelet count of 119. The bicytopenia was similar to her baseline hematologic parameters from 3 weeks back when SLE was diagnosed. The ESR was 35 mm in the 1st hour and C-reactive protein was 0.2 mg/L. Urine protein creatinine ratio at this time was 8.94 mg/g, serum albumin was 1.5 g/dL and serum creatinine was 3.44 mg/dL.

SLE Disease Activity Index was calculated to be 5, which was consistent with active disease. Management was commenced for a relapse of nephrotic syndrome and acute kidney injury (AKI) with increased steroid dosing and administration of intravenous diuretics and albumin.

Fever persisted for 8 days. On days 6 and 7 of fever, the platelet count remained static above 100. However, on day 8 of fever, her platelet count started demonstrating a downward trend with counts below 100, along with a new-onset lymphopenia. Hemoglobin remained static. The blood picture showed evidence of a viral infection. Due to the persistently dropping platelet counts and lymphopenia, despite escalation of immunosuppressive therapy, an alternate cause for thrombocytopenia apart from lupus was sought. NS1 antigen was done on day 9 of fever to test for DF. It was reported positive.

Despite the presence of 9 days of fever, the day of DF/DHF was taken as day 3–4 at the time of NS1 Ag positivity, considering the maximum duration NS1 remains in circulation. The initial fever was attributed to the SLE flare and DF was concluded to have developed as an unrelated subsequent event due to NS1 positivity.

The presence of already significant ascites and pleural effusions with hypoalbuminemia as part of nephrotic syndrome, confounded the identification of the onset of the critical phase. The onset of leakage was timed to be at the point of settling of fever with a drop in the platelet count to less than 100. The leakage was therefore, timed to be on the 3rd day of DF and the baseline PCV was 24%.

The total fluid quota of 4600 ml in 48 h was theoretically allowed for this patient after consultation with the nephrology team. But a more restrictive input was given so as not to worsen the extravascular fluid retention, while going through a nephrotic flare and AKI. Therefore, the allowed hourly input was 80 ml/h. The intravenous loop diuretic (furosemide) previously prescribed for the nephrotic syndrome was continued during the critical phase, due to the significant extravascular fluid accumulation with hypertension. Intravenous albumin was given additionally for maintenance of oncotic stability. The patient was closely monitored for impending shock in DHF due to the restrictive fluid approach and use of diuretics.

Significant events in the ascending limb were the development of intermenstrual bleeding associated with a PCV drop to 18.8% and a hemoglobin drop from 7.7 g/dL to 6.3 g/dL which required the transfusion of 1 unit of blood at the 23rd hour. She remained hemodynamically stable during this period and her routine antihypertensive medications were continued. A blood picture done at this time due to dropping hemoglobin and platelets, excluded microangiopathic hemolytic anemia (MAHA).

A venous blood gas was done to look for acidosis, as acidosis could potentially worsen hemodynamic instability in DHF. This revealed a high anion gap metabolic acidosis compatible with the ongoing AKI, and was corrected with intravenous sodium bicarbonate.

In the descending limb, at the 35th hour, there was a bedside PCV rise of > 20% (from 18.8 to 32%) without hemodynamic instability. Assuming a miscalculation of the onset of leakage and the actual timing presumed to be close to the peak of DHF, fluid bolus of 250 ml was given with gradual tailing off. Three hours following this, the patient developed desaturation and started coughing up pink frothy sputum indicating fluid overload secondary to the fluid bolus. Further supporting this, a bed side ultrasound scan revealed a distended inferior vena cava and B-lines in the lungs suggestive of pulmonary edema. Due to the pulmonary edema and consequent type 1 respiratory failure, she was intubated and ventilated. During these events, the patient remained hemodynamically stable and urine output was maintained at 1 ml/kg/h.

Intravenous diuretics were continued for the pulmonary edema. But, as the serum creatinine was noted to be rising during the critical phase from 3.45 mg/dL to 5.28 mg/dL, the patient was initiated on continuous renal replacement therapy (CRRT) to treat nephrogenic pulmonary edema.

The total amount of fluid used for the critical phase was 3150 cc.

During the course of the critical phase, her investigations were as follows (Table 1):

**Table 1:** Summary of investigations of the patient

	Day 6 (of fever) (Presentation to hospital)	Day 7	Day 8 (Day 3–4 of DF) (Onset of critical phase)	Day 9 NS1 positive Ascending limb	Day 10 Peak of critical phase	Day 11 Descending limb	Day 12 End of critical phase	Day 13 Convalescence	Day 15
**WBC** (x 1000/uL)	9.28	8.16	4.88	5.71	4.96	9.78	8.28	7.7	
**N**		5.71	3.91	4.21	3.35	7.14	6.85	6.54	
**L**	1.92	2.05	0.79	1.32	1.21	2.06	1.06	0.77	
**Hb** (g/dL)	7.7	7.7	7.7	6.7	6.3	9	8.5	8.2	
**HCT** (%)	24	22.5	23.8		18.8	26.4	25.2	25.1	
**Platelet** (x1000/uL)	119	128	83	48	70	69	76	103	142
**Cr** (mg/dL)	3.44	3.45				5.28	5.74	4.6	
**AST** (U/L)	11		11	11			49		
**ALT** (U/L)	16		25	26			51		
**CRP** (mg/dL)	0.2			1.5					

WBC showed an upward trend, despite a few fluctuations. The lymphopenia recovered. Platelets too showed an initial downward trend, then started rising during the descending limb. Hemoglobin dropped close to the peak and rose with the transfusion of one pint of blood.

During the convalescence, the patient remained intubated for 36 h post- critical phase. She remained hemodynamically stable and was continued on continuous renal replacement therapy till extubation.

**Fig. 1:**
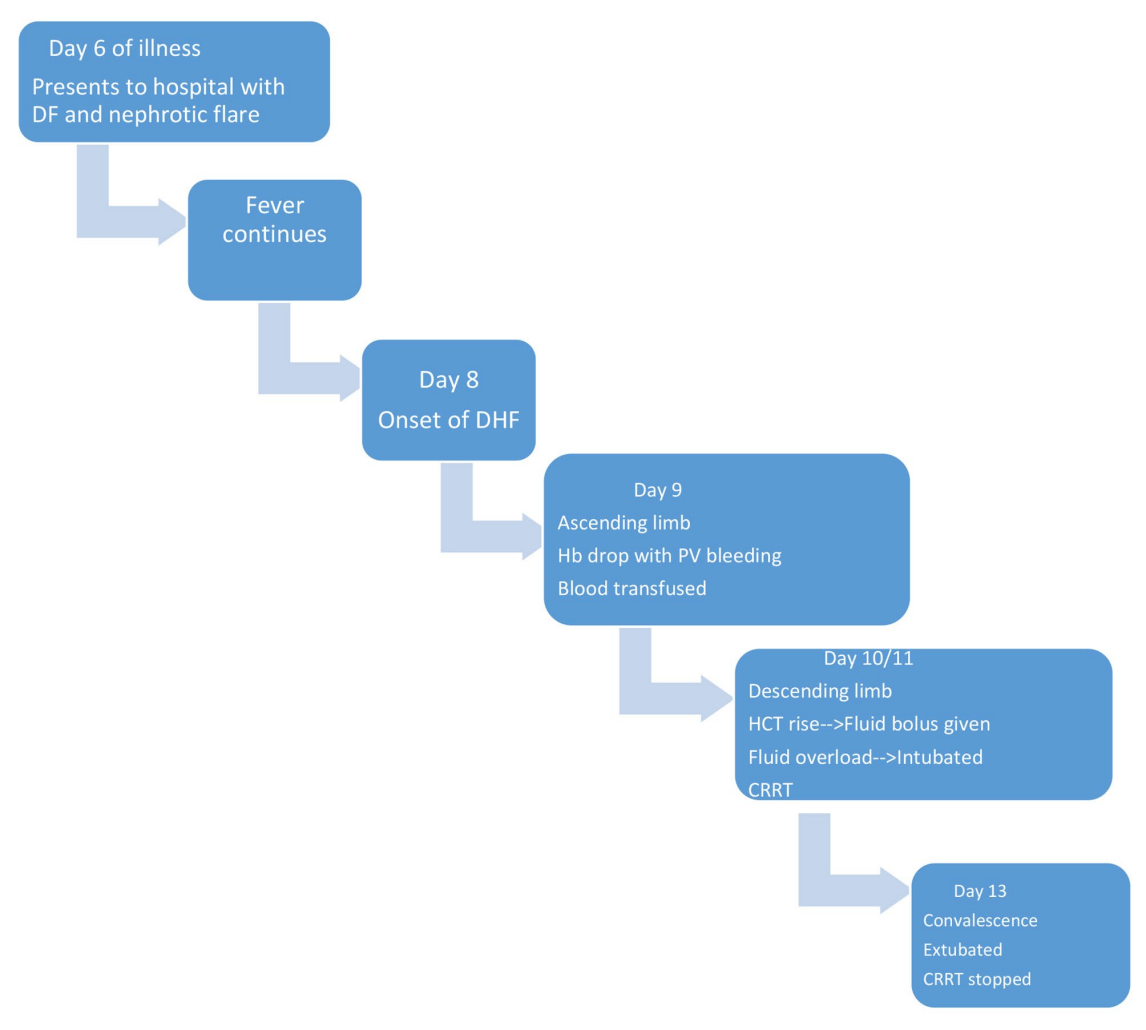
Timeline of important clinical events

Figure 1 outlines the sequence of clinical events.

## Discussion

**Fig. 2:**
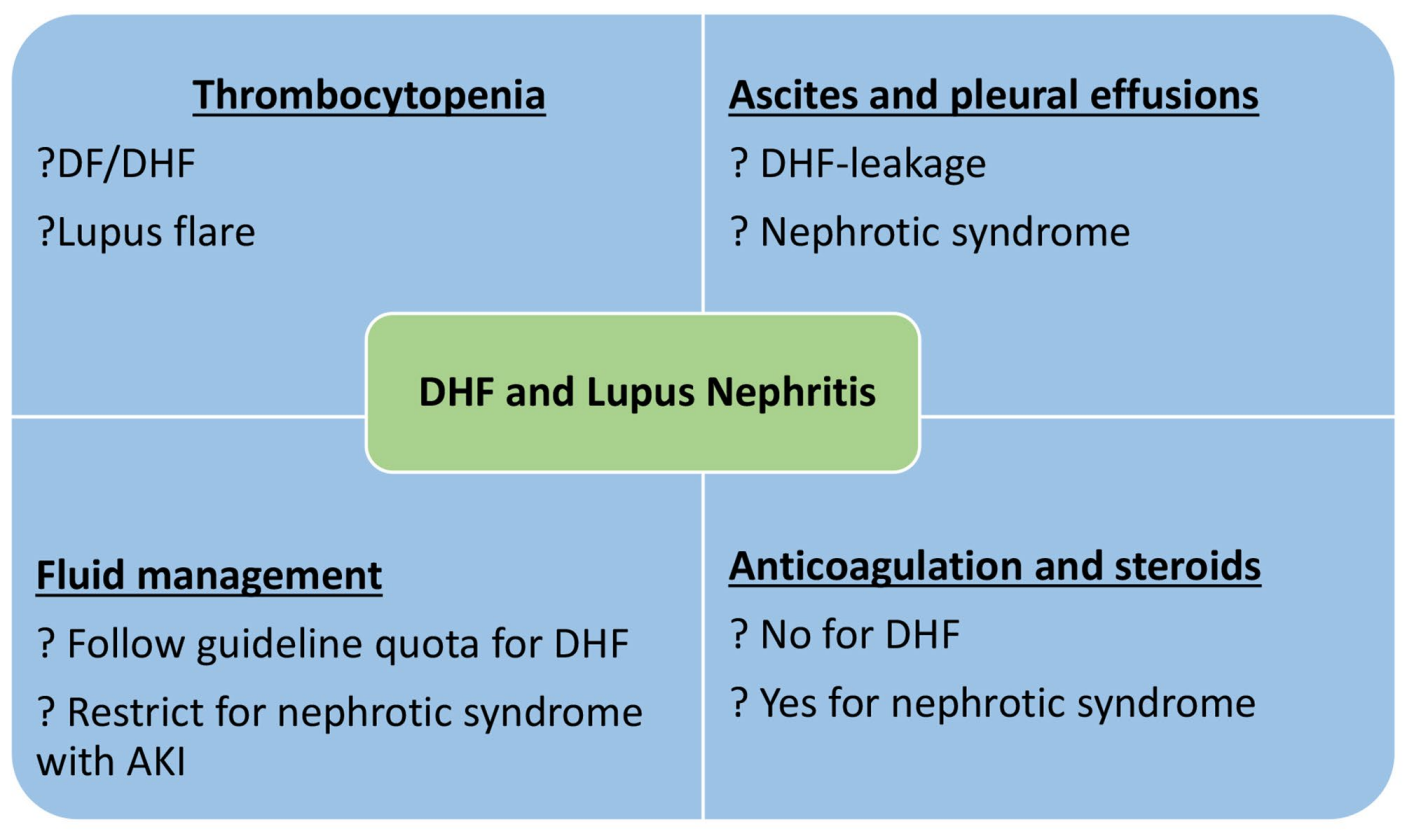
Conceptualization of clinical problems

This case report highlights the progress of a patient with DHF with preexistent hematological distortion and renal impairment that posed a unique set of diagnostic and therapeutic challenges. Specifically, there were challenges in terms of treating with fluids, steroids and anticoagulation in this patient. A discussion of five clinical problems encountered during the management of this patient will ensue below (Fig. 2).

The first was the diagnosis of DF on a background of already existent bicytopenia (anemia and thrombocytopenia) related to active SLE. Causes initially considered for bicytopenia were lupus flare and MAHA. But the worsening thrombocytopenia despite escalation of immunosuppression, and exclusion of microangiopathic hemolytic anemia on a blood picture, necessitated the consideration of an alternate cause for bicytopenia. DF/DHF was considered due to its endemicity.This patient has previously not had a dengue infection, and the time of presentation coincided with a dengue epidemic in the country. NS1 positivity further confirmed clinical suspicion of DF/DHF. The specificity of NS1 for DF is more than 99% [2]. Cytopenias in DF occur due to suppression of the bone marrow due to viral infection [3]. An additional mechanism of thrombocytopenia is due to peripheral destruction, by way of anti-platelet antibodies, apoptosis and complement lysis [3]. Another possibility for this clinical picture with extravascular fluid accumulation and bicytopenia is systemic capillary leak syndrome (SCLS), especially given the recent treatment with cyclophosphamide [4]. However, the high specificity of NS1 antigen for dengue and rarity of cyclophosphamide as a precipitant for SCLS made this unlikely [4].

The second clinical problem was identification of the onset of the critical phase in this patient. Identifiers of the critical phase as per the national dengue management guideline are progressively rising hematocrit, presence of pleural effusions and ascites, hypoalbuminemia and hypocholesterolemia, and further supporters are leukopenia and with relative lymphocytosis and thrombocytopenia less than 100 [5]. All of the above were unreliable in this patient due to the superadded lupus nephritis and per vaginal bleeding. But transition from febrile to afebrile status without clinical improvement and tenderness in the right hypochondrium too suggest DHF as per the guideline [5]. These criteria were therefore used to detect DHF in this patient. The onset of leakage was timed at the point of settling of fever with a platelet drop below 100.

The third clinical problem was management of fluid therapy in this patient: balancing the fluid management of DHF with that of NS, and management of fluid overload during descending limb with an ongoing AKI are discussed separately.

Plasma leakage in DHF occurs due to increased capillary permeability secondary to virus-related immune dysregulation [5]. The leakage is time restricted and lasts a maximum of 48 h following which resorption occurs [5]. The pathogenesis in NS also results in extravascular fluid leakage due to hypoalbuminemia. But as fluid leakage is mainly driven in adults by an overfill mechanism, the risk of hemodynamic instability is less in NS. Consequently, there is an allowance for more restrictive fluid replacement.

The presence of two pathologies that lead to intravascular volume depletion, was a risk factor for hemodynamic instability in this patient during the ascending limb. Conversely, during the descending limb, AKI was a risk factor for nephrogenic edema and fluid overload.

The guideline-recommended fluid quota for 48 h in DHF is the maintenance fluid combined with 5% of deficit [5]. It is aimed at maintaining just enough perfusion to vital organs, as extra fluid will lead to fluid overload during the descending limb. This amounted to 4600 ml in this patient, but it was decided to administer fluids more restrictively due to NS and AKI. The anticipated risk with restrictive fluid replacement combined with the use of diuretics during the ascending limb was the possibility of dengue shock. This was watched for with close monitoring of vital parameters and hematocrit. In retrospect, the patient proved to be a mild leaker, as hemodynamic stability was maintained with this restrictive approach. The authors suggest that a different approach without restriction may need to be considered for more severe leakers. However, it is interesting to note that patients with SLE have been hypothesized to have only mild dengue infection due to neutralization of the dengue virus with various types of antibodies found in SLE patients [6]. If this is in fact the case, perhaps a restrictive fluid approach could be tried on all dengue patients with lupus nephritis, but it is difficult to give general instructions without detailed studies in this regard.

With regard to management of fluid overload, loop diuretics are the mainstay of management. However, in this case, due to the ongoing AKI with rising serum creatinine, this patient was managed with both diuretics and CRRT. The transient increase in hourly input precipitated nephrogenic pulmonary edema in this patient who was already on diuretics. It was therefore, reasonably concluded that resorption of the extravasated fluid during recovery from DHF may further worsen pulmonary edema and precipitate heart failure in this patient with renal impairment incapable of adequate diuresis. It was therefore decided to empirically use CRRT in this patient. While large-scale studies are needed to support this approach, it has been successful in certain individuals [7] and another case report speculates that early use of CRRT may prevent death due to DHF [8].

The fourth clinical problem was balancing the bleeding tendency of DHF with the thrombotic risk of nephrotic syndrome. As per the national guideline, bleeding in DHF is managed with tranexamic acid with the addition of norethisterone can be added for per vaginal bleeding and blood needs to be transfused during a significant drop in the hematocrit [4]. Tranexamic acid is a fibrinolytic inhibitor and its use in DHF is not supported by adequate evidence [9]. Given the lack of compelling evidence, and the thrombotic risk of NS, tranexamic acid was avoided in this patient. Thromboprophylaxis for NS is usually provided with low molecular weight heparin, but its use is contraindicated with platelets below 50 × 10 9/L, such as in this patient [9]. As a result, thromboprophylaxis was provided with the use of graded compression stockings and pneumatic compression.

The fifth clinical problem was balancing the pros and cons of the use of steroids in this patient. The need for steroids in this patient was due to active SLE with NS, but the risk of steroids was increased risk of bleeding in DHF. The benefit was considered greater than the risk given the involvement of a major organ with SLE and steroids were continued. The bleeding was limited to per vaginal bleeding in this patient and was able to be managed with norethisterone and blood transfusion.

## Conclusions

DHF and lupus nephritis class IV are two life-threatening conditions, which are challenging to manage individually, let alone in combination. There is a dearth of guidelines pertaining to the management of dengue in the context of renal disease. While the case highlighted above cannot be generalized, it is important that challenging cases along with lessons learned therein, are shared.

The authors would like to highlight the importance of having a high suspicion of dengue in endemic areas and the importance of having a multidisciplinary approach to guide fluid therapy in challenging cases.

## Data Availability

All data generated or analyzed during this study are included in this published article [and its supplementary information files].

## List of abbreviations

AKI: Acute kidney injury
CRRT: Continuous renal replacement therapy
DF: Dengue Fever
DHF: Dengue Haemorrhagic Fever
IV: Intravenous
MAHA: Microangiopathic haemolytic anemia
NS: Nephrotic syndrome
SLE: Systemic Lupus Erythematosus
WBC: White blood cell
